# Sepsis in the emergency department: a dual challenge of early management and antimicrobial stewardship

**DOI:** 10.1186/s12879-025-11947-7

**Published:** 2025-11-12

**Authors:** Romina Corsini, Giulia Marini, Marta Ottone, Paolo Giorgi Rossi, Ivana Maria Lattuada, Valentina Cocchi, Davide Maria Francesco Lucchesi, Benedetta Cacciamani, Sara Beneventi, Samuele Cantergiani, Federico Romani, Mattia Simion, Giuseppe Russello, Sergio Mezzadri

**Affiliations:** 1 Infectious Disease Unit, Azienda Unità Sanitaria Locale-IRCCS di Reggio Emilia, Reggio Emilia, Italy; 2 Epidemiology Unit, Azienda Unità Sanitaria Locale-IRCCS di Reggio Emilia, Reggio Emilia, Italy; 3 Department of Emergency Medicine, Azienda Unità Sanitaria Locale-IRCCS di Reggio Emilia, Reggio Emilia, Italy; 4 Infectious Disease Unit, University of Modena and Reggio Emilia School of Medicine, Modena, Italy; 5 Clinical Microbiology Laboratory, Azienda Unità Sanitaria Locale-IRCCS di Reggio Emilia, Reggio Emilia, Italy

**Keywords:** Sepsis, Antimicrobial stewardship, Emergency department

## Abstract

**Background:**

Sepsis is a life-threatening condition responsible for millions of deaths worldwide. Although mortality rates have declined, sepsis remains a major global health concern. Early recognition and prompt, appropriate treatment are essential for improving patient outcomes. However, implementing effective sepsis management protocols in Emergency Departments (EDs) is often hindered by staff shortages and high patient volumes. Strengthening sepsis care is also crucial for promoting responsible antibiotic use. This study aimed to evaluate the impact of an antimicrobial stewardship (AMS) program—based on stakeholder training, audit, and feedback—implemented in the ED on the management of sepsis.

**Methods:**

A retrospective pre-post intervention analysis was conducted to assess changes in adherence to sepsis management guidelines and clinical outcomes following the introduction of the AMS program. The effect of the intervention on key process indicators was evaluated using Interrupted Time Series (ITS) analysis and logistic regression models adjusted for age and sex.

**Results:**

We analysed data from 577 septic patients in the pre-intervention period and 502 in the post-intervention period. Following implementation, sepsis recognition increased from 47.5% to 61% (*p* < 0.0001). Significant improvements were also observed in the collection of blood cultures (from 20.5% to 42.4%, *p* < 0.0001), antibiotic administration (25.7% to 46.8%, *p* < 0.0001), and crystalloid fluid administration (53% to 72%, *p* < 0.0001). Complete execution of the sepsis treatment bundle increased substantially post-intervention (OR 2.99, *p* < 0.0001). Adherence to institutional antibiotic guidelines also improved (OR 4.54, *p* < 0.0001). ITS analysis confirmed improvements in all components of the sepsis bundle and in sepsis recognition after the intervention. Adherence to local antibiotic guidelines alone (OR 0.59, 95% CI 0.33–1.04) and in combination with sepsis recognition (OR 0.51, 95% CI 0.28–1.01) was associated with a reduction in 14-day mortality.

**Conclusion:**

The AMS program led to substantial improvements in nearly all key process indicators for sepsis management, supporting the objectives of the WHO Global Action Plan on Antimicrobial Resistance, particularly in countries with high prevalence of multidrug-resistant bacteria (WHO. Global Action Plan on Antimicrobial Resistance; World Health Organization: Geneva, Switzerland; 2015). Process indicators were confirmed to be associated with improved short-term survival.

**Clinical trial number:**

Not applicable.

**Supplementary Information:**

The online version contains supplementary material available at 10.1186/s12879-025-11947-7.

## Background

Sepsis is a life-threatening organ dysfunction caused by a dysregulated host response to infection [1]. Sepsis and septic shock are major healthcare problems that impact millions of people around the world each year [2–4]. In 2017, 48.9 million cases of sepsis were recorded globally, with 11 million sepsis-related deaths accounting for nearly 19.7% of the total deaths. Mortality rates have shown a declining trend attributable to advancements in the case fatality rate and a reduction in incidence from 1990 to 2017; nevertheless, it remains a high-mortality condition [5]. Early identification and treatment of sepsis are the cornerstones for improving sepsis outcomes, as indicated in the latest guidelines from the Surviving Sepsis Campaign in 2021 [6].

Guidelines recommend the use of clinical parameter-based scores and initiating resuscitation bundles within the first 3–6 h of recognising sepsis and septic shock. The bundle includes blood culture prior to the initiation of antibiotic therapy, empirical antibiotic therapy, lactate level measurement and rapid infusion of crystalloids in hypotensive patients. Antibiotic selection should be based on local epidemiology and updated resistance probabilities. However, poor adherence to guidelines has been reported, with the difficulty of implementing individualised therapies being a major obstacle. Staff shortages and the high patient volume in the emergency department (ED) represent additional significant obstacles to the implementation of sepsis bundles [7–9].

The ED is an interface between hospitals and communities, where a significant number of antibiotic courses begin., For these reasons, it represents a crucial setting for addressing antibiotic stewardship programme (ASP) implementation [10].

Approximately 10% of ED visits are infection-related [11], ranging from common viral infections to life-threatening sepsis. The rates of adherence to local empirical antibiotic treatment guidelines in the ED are similar to those in the inpatient context: approximately 40–60% [12, 13]. ED presents many challenges for ASPs because of fast pacing, high patient and staff turnover, and the absence of feedback on patient outcomes.

Despite the recent shortage of high-quality scientific studies on antimicrobial stewardship interventions in the ED, it has been documented that multifaceted interventions, the publication of local guidelines and educational approaches aimed at changing prescribing practices have a concrete impact on antibiotic prescription in the ED [14].

In this study, we describe the impact of an antimicrobial stewardship intervention implemented in the Emergency Department of a 1,500-bed hospital, which recorded 315 emergency encounters per 1,000 inhabitants in 2022, with 6% classified as non-deferrable urgencies. We compared process indicators, including infection diagnosis and antimicrobial care, and outcome indicators of sepsis that are routinely collected in the ED before and after the intervention.

## Methods

Since November 1st, 2021, we have implemented an antimicrobial stewardship program in the ED to improve adherence to the SSC bundle and sepsis outcomes [6]. A key component of our programme was the integration of the National Early Warning Score 2 (NEWS-2) [15] into the five-level colour-coded triage system [16, 17], the establishment of 24-hour–7-day infectious disease consultant availability, ongoing education for both ED nurses and physicians, analysis of behavioural and organizational barriers to be addressed, measurement of sepsis bundle compliance, patient outcome assessment through an audit and a feedback initiative. Findings from the process indicator analysis were communicated to all ED staff via infographics (see supplementary).

### Study design and setting

We retrospectively conducted a before/after analysis comparing two cohorts, including all consecutive patients who presented at the ED with sepsis during two periods: before and after antimicrobial stewardship program implementation. The study was approved by the local Institutional Ethics Committee (Comitato Etico AVEN), which deemed the study exempt from human subject review because the data were used for their primary scope within a quality improvement and audit program and waived the need for informed consent.

### Selection of participants

**Fig. 1:**
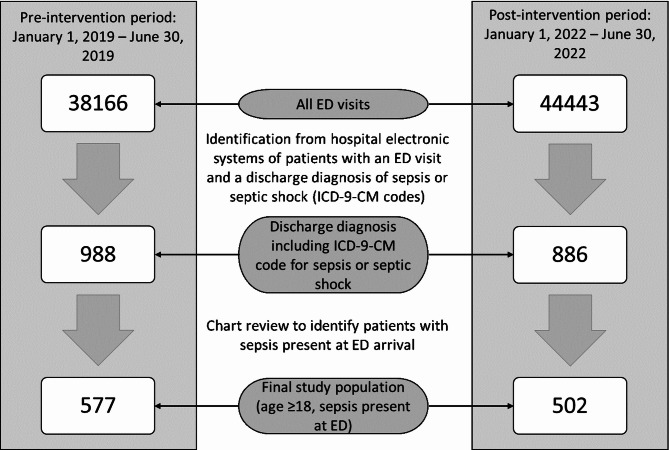
Flowchart of patients’ selection process

We retrospectively identified patients diagnosed with sepsis based on the ICD-9-CM code assigned at hospital discharge, in accordance with the definition proposed by Singer et al. [1]. We included patients with community-acquired sepsis already present at the time of admission to the emergency department (ED), confirmed through retrospective review of clinical records made by physician researchers, according to SEPSIS-3 criteria [1] (Fig. 1). Two distinct time periods were analysed: the pre-intervention phase (January 1, 2019 – June 30, 2019) and the post-intervention phase (January 1, 2022 – June 30, 2022). We did not collect data during the COVID-19 period because of alterations in the ED’s operational framework throughout the pandemic.

### Measurements

Laboratory data and clinical parameters were extracted electronically via the emergency department management program and data warehouse. Clinical data were extracted from the medical records by three independent reviewers using a standardized data collection form with predefined definitions for each variable. In case of discrepancies, consensus was reached through joint review and discussion. Although no formal inter-rater agreement was calculated, discrepancies were infrequent and resolved consistently.

### Statistical analysis

Continuous variables are reported as medians and interquartile ranges, and categorical variables are reported as proportions. The effects of the intervention on process indicator trends were evaluated through interrupted time series (ITS) analysis, and odds ratios (ORs) and 95% confidence intervals (95% CIs) were estimated via logistic models adjusted for age and sex.

ITS analysis was applied in the twelve observed months: from January to June 2019 (pre-intervention period) and from January to June 2022 (post-intervention period).

This approach uses a regression model for time series and estimates the effect of the intervention by considering trends in the 2 periods for a single treatment group [18–20].

$$\text{Yt} = {\rm \beta}{0} + {\rm \beta}{\rm 1Tt} +{\rm \beta}{2\rm Xt} + {\rm \beta}{3\rm XtTt} +\_t$$

Yt is the aggregated outcome variable (proportions) measured at each equally spaced time point t (month), Tt is the time since the start of the study, Xt is a dummy (indicator) variable representing the intervention (0 pre-intervention, 1 post-intervention period), and XtTt is an interaction term. β0 represents the intercept or starting level of the outcome variable, β1 represents the slope or trajectory of the outcome variable until the introduction of the intervention, β2 represents the change in the level of the outcome that occurs in the period immediately following the introduction of the intervention (compared with the counterfactual), and β3 represents the difference between the pre-intervention and post-intervention slopes of the outcome. Thus, we look for an effect in β2 to indicate an immediate treatment effect or in β3 to indicate a treatment effect over time [21].

We assessed whether the association between sepsis recognition, antibiotic administration and adherence to local guideline-based antibiotic therapy and 14-day mortality was appreciable in our setting. ORs with relative 95% CIs have been estimated through logistic regression models adjusting for age and sex.

As this was a retrospective observational study, the sample size was not determined a priori but was defined by the total number of eligible patients during the study period and we did not pre-define the minimal difference of interest for each relevant outcome to compute the power of the study, therefore, a formal sample size calculation was not applicable. Consistently, we did not fix a significance threshold to reject the null hypothesis and no formal test of hypothesis were performed. Furthermore, P values are reported as continuous variables and confidence intervals should be interpreted as a measure of the uncertainty of the point estimate and not as a test of hypothesis [22].

All analyses were conducted via STATA version 17 (StataCorp. 2021, Stata Statistical Software: Release 17, College Station, TX: StataCorp LLC).

## Results

### Characteristics of the study subjects

A total of 38,166 patients presented to the ED during the pre-intervention period, and 44,443 presented to the ED during the post-intervention period. We included 1079 septic patients evaluated in the ED who met the inclusion criteria during the two-phase study period: 577 patients in the pre-intervention period and 502 in the post-intervention period. Across the whole sample, 49.8% of the patients were male and the median age was 81 years.

Baseline characteristics, including physical parameters and laboratory results, were similar between the two groups (Table 1). Statistical differences were observed in, platelet count, body temperature and O2 saturation.

**Table 1:** Demographic, clinical, and laboratory patient’s characteristics

	Group 0 (*n* = 577)		Group 1 (*n* = 502)		*p*-value
	Values	Missing (*n*)	Values	Missing (*n*)	
Age, median (IQR)	81 (71–88)	0	81 (71–88)	0	0.8110
Female gender n. (%)	284 (44.71)	0	258 (51.39)	0	0.4760
SBP, median (IQR), mmHg	120 (40	72	118 (100–135)	15	0.4492
DBP, median (IQR), mmHg	70 (60–80)	71	70 (60–76)	15	0.4289
Heart rate, median (IQR), bpm	95 (78–110)	127	90 (80–106)	38	0.1667
Respiratory rate, median (IQR), breaths per minute	24 (19–30)	412	24 (20–26)	286	0.0719
Body temperature, median (IQR), °C	37.3 (36.5–38.2)	159	37.5 (36-38.2)	29	0.0072 *
O2 saturation, median (IQR), %	94 (91–97)	107	95 (91.5–97)	15	0.0133 *
Bilirubin, median (IQR), mg/dl	0.7 (0.5–1.1)	39	0.7 (0.5–1.1)	76	0.8087
Creatinine, median (IQR), mg/dl	1.24 (0.94–1.89)	17	1.36 (0.94–2.06)	6	0.1834
Leukocytes, median (IQR), x1000cell/ul	11.81 (7.88–16.47)	0	12.02 (7.81–17.85)	3	0.2857
Platelets, median (IQR), x1000cell/ul	211 (149–293)	0	204 (138–278)	3	0.0345 *
pH, median (IQR)	7.44 (7.40–7.49)	208	7.43 (7.38–7.47)	220	0.1556
CRP, median (IQR), mg/dl	12.35 (5.19–19.73)	14	10.96 (4.67–18.90)	8	0.1566
PCT, median (IQR), mg/dl	2.37 (0.67–10.18)	91	1.99 (0.62–9.08)	88	0.5513
PaO2/FiO2, median (IQR), mmHg	287 (238–336)	307	290 (228–353)	220	0.6233
GCS score, median (IQR)	15 (14–15)	499	15 (15–15)	422	0.3940

We also identified risk factors for multidrug-resistant organisms (MDROs) infections for the patients for both pre-intervention and post-intervention periods, and the results are summarized in Table 2. Among the total microbiological isolates from blood cultures during the entire period, 20% were identified as MDROs, particularly ESBL-producing Enterobacteriaceae.

**Table 2:** MDRO risk factors for patients of the two groups

	Group 0 *n*. (%)	Group 1 *n*. (%)	*p*-value
90 days previous treatment with piperacillin/tazobactam, fluoroquinolones or cephalosporins	162 (28.1)	148 (29.5)	0.6417
Previous MDRO infection	61 (10.6)	38 (8.5)	0.1123
Hospitalization in the previous 6 months	258 (44.7)	203 (40.4)	0.1756
Haemodialysis patient	15 (2.6)	20 (4.0)	0.2600
Haematological oncology patient	117 (20.3)	106 (21.1)	0.7791
Nursing home resident patient	117 (20.3)	75 (14.9)	0.0295*
Patient with device	125 (21.7)	109 (21.7)	1.0000
Monoclonal antibody therapies in the previous 6 months	10 (1.7)	10 (2.0)	0.9297
CRE colonization	12 (2.0)	7 (1.4)	0.5317
Any combination of 2 risk factors	260 (45.1)	216 (43.2)	0.5638

### Main results

**Fig. 2:**
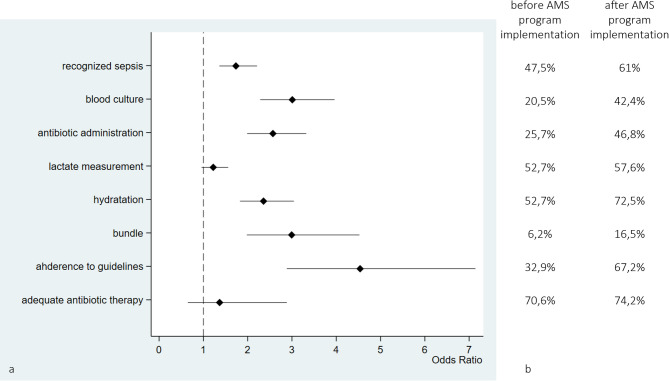
Forest plots of process indicators for the AMS program illustrating compliance with sepsis bundle and evaluation of appropriateness and adequacy of antibiotic therapy. **b**) % of the process and antibiotic therapy indicators before and after the implementation of the AMS program

Following the implementation of the AMS program, there was a notable improvement in sepsis recognition by ED physician, increasing from 47.5% to 61%, with an OR of 1.73 (p-value < 0.0001). With respect to sepsis bundle elements, there was a substantial increase in blood culture sampling (from 20.5% to 42.4%, OR 3.01, p value < 0.0001) with similar percentage of negative test (45.8% for pre-intervention period and 44.6% for post-intervention), antibiotic administration (from 25.7% to 46.8%, OR 2.57, p value < 0.0001) and crystalloid administration (from 53% to 72%, OR 2.36, p value < 0.0001), whereas lactate measurements showed a small change. The comprehensive execution of the sepsis bundle demonstrated a noteworthy increase in the post-intervention period (OR 2.99, p value < 0.0001) (Fig. 2; Table 1 supplementary).

We also investigated adherence to our institutional guidelines on antibiotic treatment, which improved from 33% to 67% (OR 4.54, p value < 0.0001). The adequacy of the antibiotic, assessed according to blood culture antibiogram coverage, improved from 70% to 74% after AMS program implementation; nevertheless, the change was compatible with random fluctuations.

**Fig. 3:**
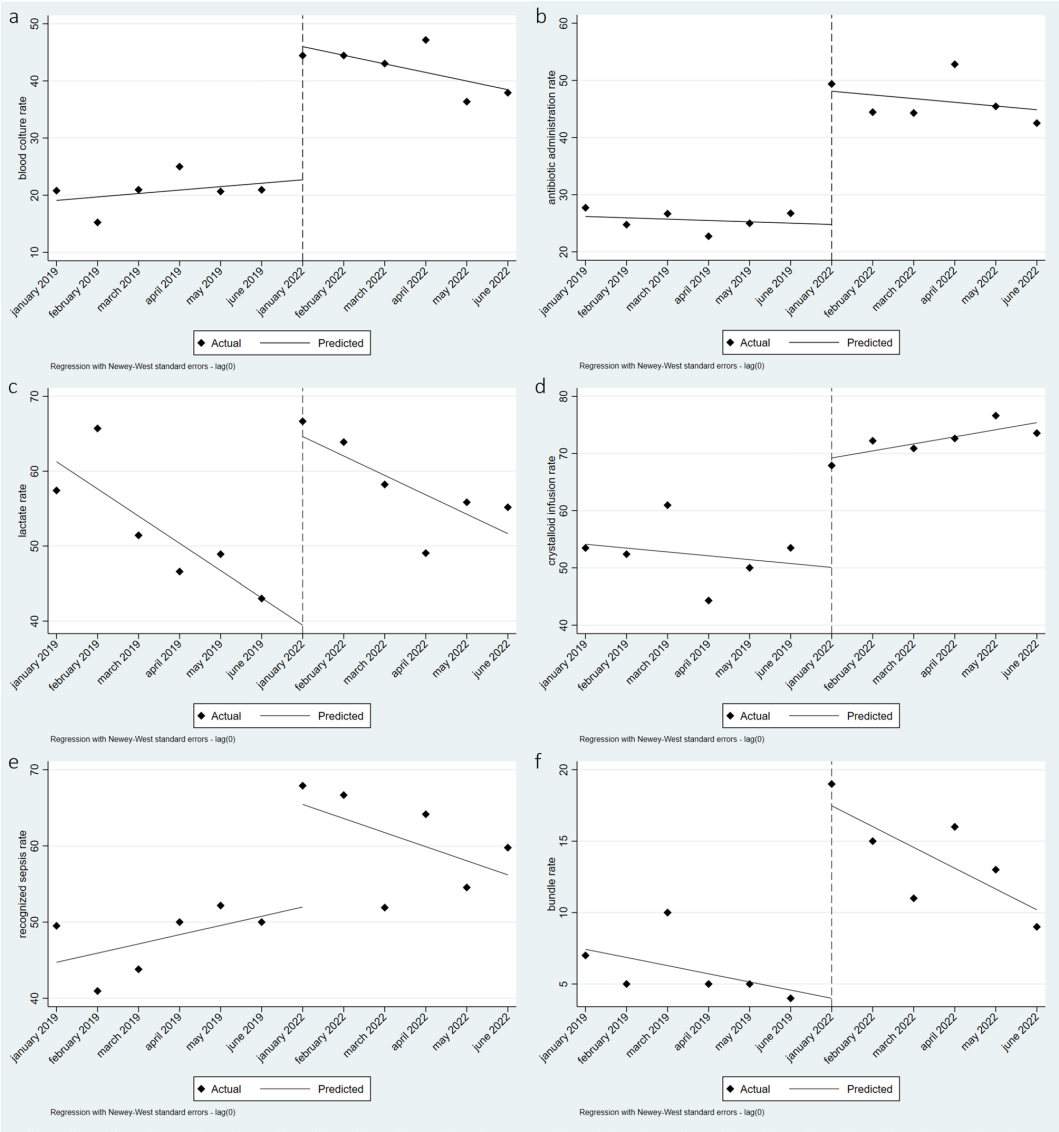
Effect of AMS program implementation on **a**) blood culture rate; **b**) antibiotic administration rate; **c**) lactate measurement rate; **d**) crystalloid infusion rate; **e**) sepsis recognition in ED and **f**) bundle accomplishment. The solid lines represent the estimated slopes by the regression model. Dashed lines represent the beginning of the post-intervention phase

The ITS analysis (Fig. 3) revealed a noteworthy improvement in the rates of all elements within the sepsis bundle and sepsis recognition at the beginning of the post-intervention study phase (Table 3, ß2 level post-intervention).

**Table 3:** Results of ITS analysis comparing the sepsis bundle elements and sepsis recognition in ED in the two study phases

	Β1 trend baseline (IC 95%)	*p* value	β2 level post intervention (IC 95%)	*p* value	β3 trend post intervention (IC 95%)	*p* value
Blood culture	-0.60 (-0.82; 2.02)	0.358	23.3 (17.9; 28.7)	0.000*	-2.10 (-4.0; -0.26)	0.030*
Antibiotic administration	-0.23 (-1.24; -0.77)	0.609	23.3 (17.08; 29.57)	0.000*	-0.42 (‘-2.16; 1.32)	0.596
Lactates measurement	3.64 (-6.21; -1.06)	0.012*	25.2 (17.38; 32.96)	0.000*	1.04 (-2.13; 4.22)	0.471
Hydration	-0.68 (-2.20;0.84)	0.335	19.15 (10.42;27.88)	0.001*	1.91 (-0.01;3.83)	0.051
Recognized sepsis	1.21 (-1.19;3.61)	0.279	13.48 (2.85;24.10)	0.019*	-3.06 (-6.35;0.23)	0.065
Complete sepsis bundle	-0.57 (-1.19; 0.04)	0.065	13.5 (9.5; 17.4)	0.000*	-0.89 (-2.02; 0.25)	0.110

Only minor changes in the trends of the analysed elements were observed during the six-month period following the implementation of the AMS programme, except for the blood culture sampling rate, which exhibited a negative trend of -2.10, p value of 0.030 (Table 3, ß3 trend post-intervention).

**Fig. 4:**
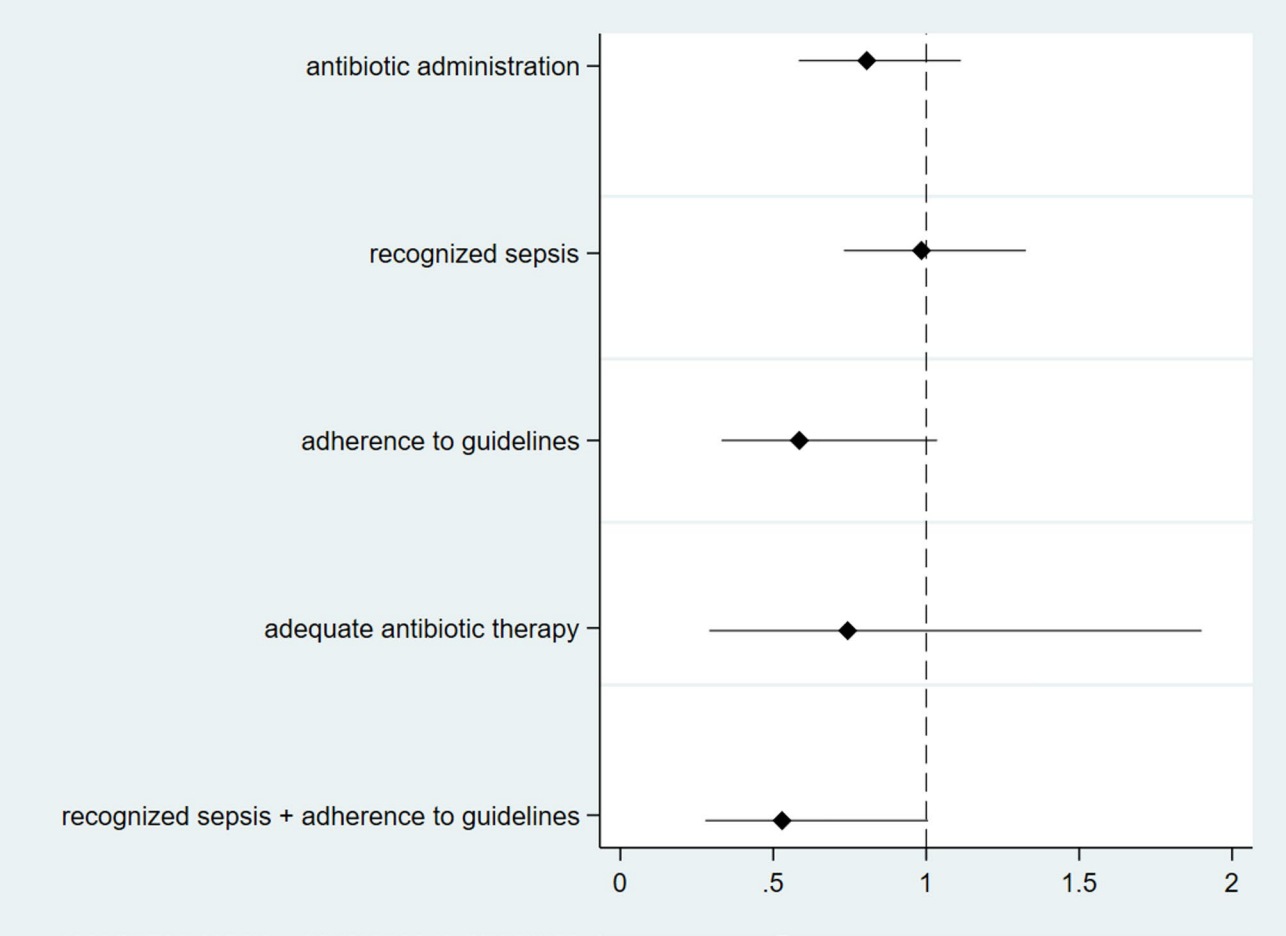
Forest plot of the association between sepsis recognition, antibiotic therapy administration and characteristics and 14 days’ mortality

We further investigated the associations between overall patient mortality at 14 days and antibiotic administration, the adequacy of antibiotic therapy, adherence to local guidelines regarding antibiotic choice, sepsis recognition and the combined impact of the latter two (Fig. 4). Adherence to local guidelines on antibiotic choice alone (OR 0.59, 95% CI 0.33–1.04) and combined with sepsis recognition (OR 0.51, 95% CI 0.28–1.01) was possibly associated with mortality at 14 days.

## Discussion

In our work, we observed a strong improvement in almost all process indicators after the implementation of the AMS program. The demographic and clinical characteristics of the two groups before and after the intervention were similar, except for platelet count, body temperature and O2 saturation; these differences were not deemed clinically significant.

Sepsis recognition, antibiotic administration and adherence to guidelines, which are key elements of AMS [6], are linked to mortality, even if the sample size is not large enough to exclude the possibility of this being due to chance. All these findings may be due to other underlying trends; the COVID-19 pandemic represents a strong inference that limits the possibility of inferring causality in our observations; nevertheless, the ITS suggests that improvements do not follow a previous trend. The overall consistency of the data suggests that the AMS program had a positive impact on process- and patient-relevant outcomes beyond the uncertainty and possible biases behind each single measure.

Implementing our AMS program demanded long-term commitment and the creation of a multifaceted team of professionals with the objective of placing the septic patient at the centre of the organizational scheme rather than an intervention with a mandated protocol [23].

Despite some heterogeneity, a meta-analysis of 50 observational studies on the effects of performance improvement programs revealed that these programs were associated with better adherence to sepsis bundles and a reduction in mortality (OR, 0.66; 95% CI, 0.61–0.72) in patients with sepsis and septic shock [24]. Consistent with prior observations, our investigation demonstrated that early recognition of sepsis, coupled with adherence to local guidelines, leads to an improvement in 14-day mortality. These data underscore the importance of consistently updating guidelines in our epidemiological context characterized by a high incidence of multidrug-resistant organisms (MDROs).

In particular, through interrupted time series analysis, we revealed a discontinuity in the process indicators after the intervention and the establishment of a new decreasing trend, highlighting the importance of ongoing education and the need to introduce process facilitation tools.

Importantly, the guidelines are an excellent reference source of evidence-based knowledge, but alone, they are unlikely to cause substantial changes in the behaviour of clinicians at the bedside.

Despite sponsorship from 30 international organizations and multiple studies supporting severe sepsis guidelines and bundles, adoption into practice has been linked with perceived and real barriers to implementation. Importantly, the time from validation to implementation of a new clinical practice guideline is typically 17 years [25].

Early adopters disseminate knowledge and proceed with the development of protocols and the implementation of a performance improvement program that involves the measurement of data and feedback [26]. Successful implementation with significant improvement in sepsis bundle compliance and a marked reduction in mortality has already been reported by early adopters of the guidelines and bundles [27].

Adopting, adapting and updating existing workflows is necessary to promote the optimal use of antibiotics in EDs, as recommended by evidence-based international guidelines such as the International Guidelines for the Management of Sepsis and Septic Shock 2021.

Moreover, our extensive intervention required the collaborative formulation of training programs designed to promote dialogue among healthcare professionals. This initiative culminated in establishing a task force capable of maintaining a continuous cycle of audits and feedback.

The procedures performed in the ED influence subsequent levels of septic patient management, making the correct approach in the early hours of care crucial. What emerges from our work and the analysis of the relevant literature [6, 28] is the lack of individual process indicators capable of impacting the primary outcome of mortality. For this reason, there is a need for a comprehensive approach involving all levels of care, including primary care and inpatient wards.

When focusing on the septic patient pathway, considerations should include the suspicion of sepsis in primary care, the adoption of a unique severity score across hospital and community settings (NEWS-2), referral to the ED, fluid management, source control, the need for de-escalation and appropriate duration of antibiotic therapy and potential pharmacological interactions in elderly and comorbid populations.

Our before-after study design presents several limitations, including temporal changes in populations and standards of care, particularly in the aftermath of the COVID-19 pandemic.

The integration of SARS-CoV-2 molecular diagnostics into clinical workflows, which rely on laboratory-based rather than point-of-care testing, increased the workload in ED and often delayed the recognition and treatment of other acute infectious conditions, such as sepsis. Another limitation is the presence of missing data for some laboratory values or physiological parameters.

In summary, our study could be generalised across different healthcare settings, focusing on: the assessment of organisational and educational needs; analysis of local epidemiology; dissemination of the NEWS2 score as a tool for objective triage and the implementation of an ongoing audit and feedback strategy. Improvements in nearly all process indicators related to ED sepsis management consistently occurred after the intervention. It is also reassuring that, in our setting, the link between process indicators and improvement of survival is confirmed with a magnitude, more than 40% mortality reduction at 14 days for adherence to guidelines, similar to that observed in experimental settings (ref). Nevertheless, ongoing training initiatives and organizational adaptations are imperative to ensure enduring improvement,

Given that our hospital is in an area with a high prevalence of MDR gram-negative bacteria, constant adaptation of local guidelines is equally important to establish appropriate empirical antibiotic therapy based on the likelihood of multidrug-resistant strains.

## Supplementary Information

Below is the link to the electronic supplementary material.

Supplementary Material 1

## Data Availability

The de-identified dataset for this study is available upon request, from the date of article publication by contacting Romina Corsini, at romina.corsini@ausl.re.it.

## Abbreviations

AMS: Antimicrobial Stewardship
ASP: Antibiotic Stewardship Programme
CRE: Carbapenem-Resistant Enterobacterales
CRP: C-Reactive Protein
DBP: Diastolic Blood Pressure
ED: Emergency Department
ESBL: Extended-Spectrum Beta-Lactamases
GCS: Glasgow Coma Scale
ICD-9-CM: International Classification of Diseases, Ninth Revision, Clinical Modification
IQR: Interquartile Range
ITS: Interrupted Time Series
MDRO: Multidrug-Resistant Organisms
NEWS-2: National Early Warning Score 2
OR: Odds Ratio
PCT: Procalcitonin
SBP: Systolic Blood Pressure
