# Supplementary material for: Sepsis in the emergency department: a dual challenge of early management and antimicrobial stewardship
Source: BMC Infect Dis. 2025 Nov 12;25:1563. doi: 10.1186/s12879-025-11947-7 (PMC12613919; doi:10.1186/s12879-025-11947-7)
Supplement: Supplementary file 1 — Supplementary Material 1 [file 12879_2025_11947_MOESM1_ESM.docx]

**Supplementary**

**Intervention description**

An antimicrobial stewardship intervention was implemented in the ED through a mandatory training program conducted between November 1, 2021, and February 1, 2022. To facilitate participation, ten training sessions were held on different dates. The training team was composed of peers, including emergency nurses, infectious diseases specialists and emergency physicians.

Anonymous surveys were administered to assess staff perceptions of team organization and the ED's capacity to manage septic patients. The sessions included a review of the latest international guidelines on sepsis management [1] and a discussion of organizational barriers to implementing the sepsis bundle. The National Early Warning Score 2 (NEWS2) was introduced as a triage tool to support early recognition of clinical deterioration.

The local epidemiological context, with a focus on multidrug-resistant organisms, was also presented. In addition, the availability of 24/7 infectious diseases consultation and an expanded formulary of antibiotics was introduced. Finally, after reviewing process indicators related to the sepsis bundle, targeted feedback was provided to ED staff through infographics and dedicated feedback meetings.

|  | Recognized sepsis | | Blood colture | | Antibiotic administration | | Adherence to  guidelines * | | Adequate antibiotic therapy ° | | Lactate | | Hydratation | | Bundle completition | |
| --- | --- | --- | --- | --- | --- | --- | --- | --- | --- | --- | --- | --- | --- | --- | --- | --- |
|  | OR (95% CI) | P value | OR (95% CI) | P value | OR (95% CI) | P value | OR (95% CI) | P value | OR (95% CI) | P value | OR (95% CI) | P value | OR (95% CI) | P value | OR (95% CI) | P value |
| age | 1.00 (0.99; 1.01) | 0.867 | 0.97 (0.96; 0.98) | 0.000 | 0.99 (0.98;1.00) | 0.011 | 0.98 (0.97;1.00) | 0.005 | 0.97 (0.94;0.99) | 0.017 | 1.02 (1.01; 1.03) | 0.000 | 1.00 (0.99; 1.10) | 0.657 | 0.99 (0.97; 1.00) | 0.029 |
| sex (female) | 1 |  | 1 |  | 1 |  | 1 |  | 1 |  | 1 |  | 1 |  | 1 |  |
| (male) | 1.18 (0.92; 1.51) | 0.182 | 1.21 (0.92; 1.60) | 0.166 | 1.03 (0.80; 1.34) | 0.795 | 0.61 (0.39;0.94) | 0.028 | 0.80 (0.40; 1.60) | 0.529 | 0.94 (0.73; 1.20) | 0.611 | 0.88 (0.68; 1.13) | 0.314 | 0.96 (0.65; 1.42) | 0.853 |
| intervention | 1.73 (1.36; 2.21) | 0.000* | 3.01 (2.28; 3.96) | 0.000* | 2.57 (1.99; 3.32) | 0.000* | 4.54 (2.88;7.14) | 0.000* | 1.37 (0.64; 2.88) | 0.413 | 1.22 (0.96; 1.56) | 0.106 | 2.36 (1.82; 3.04) | 0.000* | 2.99 (1.98; 4.52) | 0.000* |

Table 1. Logistic regression analysis to assess the impact of AMS intervention on sepsis bundle elements across all study participants (n=1079). *Evaluation of guideline adherence was limited to patients who received antibiotics in the ED (n=383) and ° assessment of antibiotic therapy adequacy was restricted to those who received an antibiotic therapy in ED with positive blood cultures and antibiograms (n=175).
